# Neighborhood social environments and mental health among youth and adults in public housing

**DOI:** 10.1002/ajcp.70036

**Published:** 2025-11-28

**Authors:** Jane Leer, Lindsay Lanteri, Rebekah Levine Coley, Samantha Teixeira

**Affiliations:** ^1^ Carolyn A and Peter S Lynch School of Education and Human Development Boston College Chestnut Hill USA; ^2^ Department of Psychology San Diego State University San Diego USA; ^3^ School of Social Work Boston College Chestnut Hill USA

**Keywords:** intergroup relations, mental health, neighborhoods, place attachment, public housing, social cohesion

## Abstract

Neighborhoods influence health in part through social processes. However, little is known about how multiple neighborhood social processes co‐occur, or about within (vs. between) neighborhood variation in social processes and health. This study asked how residents of a large public housing development describe their neighborhood and used latent profile analysis to develop profiles of residents according to their social environment perceptions (*N* = 527, *M*_age_ = 44, 15% Asian, 19% Black, 38% Latino, 19% White, 8% other). We included cross–race interactions as an understudied aspect of the social environment, along with social cohesion, place attachment, sense of safety, and neighborhood problems. Five profiles were identified. The largest (35% of participants) was generally content with their neighborhood. Another 18% had strongly positive perceptions, 18% were well connected but concerned about neighborhood problems, 15% were socially disengaged, and 15% were strongly dissatisfied. Profile membership was systematically related to individual and contextual factors. Anxiety and depression were highest in the strongly dissatisfied profile and the two profiles containing a mix of positive and negative perceptions (connected but concerned and socially disengaged). Findings show how differences within (not just between) neighborhoods relate to health and have implications for social programs targeting the unique needs and strengths of public housing residents.

## Highlights

- Residents of a single public housing neighborhood had diverse opinions about their neighborhood.
- Perceptions differed according to individual and contextual factors.
- There were large differences in mental health across social environment perception profiles.

Place matters for health. Decades of research link neighborhood factors such as concentrated poverty and social isolation to poor physical and mental health outcomes above and beyond individual demographic and socioeconomic characteristics (see Arcaya et al., 2024 for review). More recently, research has sought to unpack the “black box” of neighborhood effects to identify key mechanisms through which neighborhoods may influence health (e.g., Coley et al., 2021; Jakobsen et al., 2022; Kim, 2010; Sharkey & Faber, 2014). Social processes are one such mechanism, including stressors like disorder and violence, as well as strengths like social cohesion and feelings of attachment to the neighborhood.

Numerous studies have focused on specific social processes in isolation, but neighborhoods contain a multitude of social processes. Even within the same neighborhood, risks and strengths exist simultaneously (Teixeira et al., 2020; Walton, 2016), yet little is known about how different types of neighborhood social processes co‐occur. To address this gap, this study takes a comprehensive, person‐centered approach to identify profiles (or subgroups) of people according to their perceptions of multiple neighborhood stressors and strengths.

We focus on a single large and racially diverse public housing development, which enables us to address two other important gaps in the literature on neighborhood social processes. First, perceptions of constructs such as social cohesion and neighborhood problems are subjective and vary substantially among residents of the same neighborhood (Becker, 2019; Morenoff et al., 2001; Raudenbush & Sampson, 1999; Schmidt et al., 2014), yet little is known about what explains within (vs*.* between) neighborhood variation in these and other social processes. By focusing on a single neighborhood, we contribute to identifying individual and contextual factors that may explain why neighbors can hold such different views about the quality of their neighborhood social environment and how such views are associated with individual differences in health outcomes. Second, racial diversity in neighborhoods across the U.S. has increased markedly in the past 30 years (Mellnik & Dam, 2022), yet limited research has examined the nature of intergroup (cross‐race) contact in neighborhoods. The racial diversity of our study context makes it an ideal setting to examine intergroup contact within (not just between) neighborhoods.

## LINKS BETWEEN NEIGHBORHOOD SOCIAL ENVIRONMENTS AND MENTAL HEALTH

Research on social determinants of health shows how structural and social factors beyond the individual and outside of the traditional reach of the health care sector affect health (Phelan & Link, 2015). Neighborhoods are one example. Structural forces such as racism and classism shape neighborhood resource disparities (e.g., inequitable access to housing quality, green space, and safety), which in turn affect the neighborhood social environment and contribute to population health disparities (Phelan & Link, 2015; Rothstein, 2017).

Theoretical and empirical research identifies three broad domains of the neighborhood social environment: (1) the quality of relationships between neighbors (e.g., social cohesion, defined as interpersonal trust between neighbors and a sense of shared norms and values, Sampson, 2012); (2) social psychological feelings or emotional affect towards the neighborhood (e.g., place attachment; Giuliani, 2003, and sense of safety; Earls et al., 2007); and (3) perceptions of neighborhood problems. Positive relationships between neighbors and feelings of attachment and safety may support positive mental health by providing an emotionally supportive environment that helps residents cope with day‐to‐day stressors and encourages healthy behaviors (Jakobsen et al., 2022; Sampson, 2012). In contrast, persistent exposure to neighborhood problems like drug use and violence can lead to chronic stress, loneliness, and isolation, all of which are known to negatively impact mental health (van Deurzen et al., 2016). Indeed, past work has linked cohesion, place attachment, and sense of safety to positive mental health outcomes (Hong et al., 2014; Rios et al., 2012; Scannell & Gifford, 2017), whereas neighborhood problems have been linked to worse mental health outcomes (Browning et al., 2013; Kim, 2010). However, neighborhood social processes are complex and multifaceted. For example, individuals who feel well connected to their neighbors and strongly attached to their neighborhood may still be concerned about neighborhood problems, and the mental health implications of this mix of positive and negative perceptions of the neighborhood social environment are not well understood.

### Between‐ versus within‐neighborhood variation in social environment perceptions

Most quantitative research on the social mechanisms linking neighborhoods to health compares *between* neighborhoods to examine how structural factors (e.g., neighborhood poverty) affect health via social processes. However, previous research suggests that the vast majority (70%–90%) of the variation in social processes comes from *within*‐, rather than between‐neighborhood variation (Becker, 2019; Morenoff et al., 2001; Raudenbush & Sampson, 1999; Schmidt et al., 2014). Limited research has examined why individuals in the same neighborhood can hold such different perceptions of their neighborhood social environment.

A better understanding of within‐neighborhood variation in social environment perceptions could contribute to identifying ways to strengthen neighborhoods and potentially improve health outcomes. For example, the spatial organization of homes around communal spaces (e.g., parks, courtyards) could shape the amount of time that people spend outside getting to know neighbors, which could in turn affect social cohesion and safety (Jennings & Bamkole, 2019). Family or individual‐level factors could also explain within‐neighborhood differences in social environment perceptions. Compared to single adults, families with children may be especially concerned about neighborhood safety. Compared to men, women might be more aware of and vigilant towards potential safety threats. There also may be differences by race, as well as intersections between race and gender, though the nature of these differences may depend on the neighborhood's history of racial segregation and gendered racial power dynamics.

The existing literature's focus on between‐ versus within‐neighborhood analyses also risks emphasizing a deficit‐perspective in which high‐poverty neighborhoods are consistently associated with more neighborhood problems and fewer positive social processes. This can be stigmatizing for those residing in high‐poverty neighborhoods (Wutich et al., 2014), and limits attention to the many strengths, including strong social ties, that high poverty neighborhoods may possess. Studies examining social life in public housing neighborhoods have found close bonds between neighbors and a strong sense of community (Shamsuddin & Vale, 2017; Teixeira et al., 2020; Walton, 2016). For instance, a study of a public housing development in the Midwest found that sense of ownership (e.g., through community gardens) and symbolic representation (e.g., through displaying country‐of‐origin flags) facilitated a strong sense of community among residents (Walton, 2016). Studies like these that seek to identify strengths in high‐poverty neighborhoods provide an important contrast from the deficit lens in which such neighborhoods are portrayed as crime‐ridden, dangerous, and lacking in positive social ties (Walsh et al., 2024).

### Neighborhood intergroup contact as a critical yet underexamined aspect of neighborhoods

In addition to structural disadvantage, racial diversity is theorized to detract from neighborhood social cohesion and stir greater violence and disorder (Putnam, 2007; Sampson & Groves, 1989). People tend to socialize with members of their racial in‐group and find cross‐race communication challenging, which may hamper the formation of strong social ties in diverse neighborhoods and decrease general trust and community cohesion. Empirical evidence is inconclusive, however, with some studies supporting this theory (e.g., Alesina & La Ferrara, 2000; Putnam, 2007), and others showing null or positive relations between neighborhood racial diversity and social cohesion (Gijserts et al., 2012; Schmid et al., 2014).

Moreover, there is a dearth of research on the nature and quality of intergroup (e.g., cross‐race) contact in neighborhoods. Existing evidence focuses on the direct relation between neighborhood diversity and social cohesion or trust, but the potential mechanisms are rarely examined. One study in England found that neighborhood racial diversity was associated with more frequent intergroup contact and improved attitudes towards other racial groups, which resulted in greater social trust (Schmid et al., 2014). Qualitative research in the U.S. context suggests that cross‐race contact in racially diverse neighborhoods is infrequent and marked by experiences of discrimination among racially minoritized residents (e.g., Chaskin et al., 2012, 2013; Douds, 2021; McCormick et al., 2012; Tach, 2009). However, these studies focus on neighborhoods that are racially diverse and economically mixed, which makes it difficult to disentangle the effects of racial diversity from the effects of *racialized economic inequality* on cross‐race interactions.

Cross‐race interactions may be more positive in the context of neighborhoods that are racially diverse yet economically homogeneous. Intergroup contact theory (Allport, 1954; Pettigrew, 1998) posits that exposure to racial outgroups can increase intergroup trust and reduce prejudice, but only when four conditions are met: the groups need to (1) have equal status, (2) be working towards a common goal, (3) in a cooperative context, and (4) where authorities endorse intergroup contact. Recent work examining racially diverse affluent neighborhoods found only superficial, constrained racial harmony (Douds, 2021), but the nature of cross‐race interactions on the other end of the socioeconomic continuum—that is, racially diverse high poverty neighborhoods—is less well understood. Further, similar to the broader research on neighborhood social processes, most studies compare between neighborhoods with varying levels of diversity, without attention to *within* neighborhood differences in perceptions of intergroup interactions. Within neighborhoods, perceptions of intergroup interactions are likely to vary depending on each racial groups' unique history of inclusion and exclusion in the neighborhood and surrounding social context, among other factors.

## RESEARCH AIMS

This study has three aims. First, we asked how youth and adults in a single large and racially diverse public housing development describe their neighborhood social environment and categorized these reports into profiles. Second, we examined individual and contextual correlates of within‐neighborhood variation in social environment profiles. Third, we linked neighborhood social environmental profiles to individual mental health outcomes (anxiety and depression). Whereas most research uses a variable‐centered approach to isolate the relationship between specific variables (e.g., social cohesion) and health, we employed a person‐centered approach (latent profile analysis) to identify how multiple aspects of the neighborhood social environment co‐occur. We hypothesized that there would be distinct profiles, or groups of residents with varying perceptions, but we did not have specific hypotheses regarding the number or composition of profiles. To address gaps in understanding of neighborhood intergroup contact, the social environment factors that we examined included perceptions of intergroup interaction quality, intergroup equality, social cohesion, place attachment, sense of safety, and neighborhood problems. We further hypothesized that individual and contextual factors, including demographic, economic, and housing characteristics, would explain variation in social environment perceptions, and that the profiles would relate to mental health differently depending on their balance of positive and negative perceptions of neighborhood social processes.

## METHODS

### Study context

This study is situated in a public housing development in New England, one of the oldest and largest public housing developments in the United States. In 2022, when data for this study were collected, the development included approximately 1000 households and 2,000 residents, integrated across numerous dimensions, including family structure (46% single adults; 54% families); age (29% children; 10% young adults (18–24); 44% working‐age adults (25–61); 18% older adults); and race and ethnicity (38% Latino; 27% Black; 22% White; and 13% Asian). This makes it an ideal context for our study. The combination of structural poverty and racial diversity is theorized to detract from neighborhood social processes by instilling competition for essential resource (Sampson & Groves, 1989), though empirical research reveals positive relationships do exist in such contexts (Teixeira et al., 2020; Walsh et al., 2024; Walton, 2016). The neighborhood under study is racially diverse, yet relatively economically homogeneous, in that all residents have incomes that qualify them for public housing (which typically means earning no more than 80% of the area median income, though specific income requirements for public housing are set each year by the public housing authority; HUD, 2025). In this way, we move beyond prior work on cross‐race interactions in economically mixed neighborhoods by examining cross‐race interactions controlling for class.

The broader neighborhood within which this study is situated was once home to the largest concentration of White people living in poverty in the United States, most of whom were Irish immigrants who faced discrimination in housing, labor, and education elsewhere in the city (Vale, 2007). During the 1970s, the neighborhood became known for its violent protests against court‐ordered school integration, and the housing development itself remained virtually all‐White until 1988, when a class action lawsuit forced the local housing authority to integrate the development (Dominguez, 2012). Since then, the development has become increasingly diverse, and there is some evidence that as Latino and Asian residents moved in, neighborhood racial tensions dissipated (Dominguez, 2012; Satcher et al., 2024). This history likely shapes residents' feelings towards their neighborhood and perceptions of the social environment.

### Data

Data come from Wave 1 of the Housing Opportunity and Mobility Experiment (HOME) Study, a mixed‐methods, quasi‐experimental evaluation of the effects of public housing redevelopment on resident health and wellbeing. The data used here are from individual surveys conducted before the planned redevelopment into a larger mixed‐income community. The study sample was selected to estimate the effects of the redevelopment by leveraging the phased roll‐out plan. All households included in the first phase of the redevelopment were included in the sampling frame, along with a matched comparison group of households who will remain in place without experiencing housing changes until phase two of the redevelopment. Data were collected in person (70% of the sample) and online (30%) with a response rate of 67%. In most cases, one adult per household and all youth aged 12 to 24 were recruited from each targeted household. There are a total of 555 individual participants; this analysis excludes 28 participants with missing data on all five neighborhood environment indicators for a total *N* of 527. Participants were 44 years old on average, including a very broad age range from 12 to 99 years, and majority female (66%). Reflecting the diversity of the broader development, 38% were Latino, 20% Black, 20% White, 15% Asian, and 8% Other. We use the term “Latino” to be consistent with how the community in this study self‐identifies. Among the Latino sample, 55% were Dominican, 32% Puerto Rican, and 11% reported some other Hispanic/Latino heritage (e.g., Guatemalan, Honduran). The other race category includes 42 individuals who did not identify as Latino and were either Multiracial, Native Hawaiian or Pacific Islander, American Indian, or some other race or ethnicity. Unfortunately, the cell sizes were too small to analyze these groups separately without compromising participant confidentiality and statistical power.

### Measures

#### Social environment indicators

Five measures assessed residents' perceptions of the neighborhood social environment: Neighborhood place attachment was assessed with three items designed to capture the extent to which participants felt at home in their neighborhood and consider it a good place for them to live (e.g., “you feel like you belong in this community”) based on Curley (2010). Items were scored on a 4‐point Likert scale from strongly disagree (1) to strongly agree (4), with the mean across all four items used as the composite (α = 0.86). Social cohesion was measured with four items (e.g., “people in your neighborhood can be trusted”) using a scale developed for the Project on Human Development in Chicago Neighborhoods (PHDCN; Sampson et al., 1997; 1999) that has been widely used in neighborhood research (Browning et al., 2008; Hong et al., 2014; Schmidt et al., 2014). Items were scored on a 4‐point Likert scale from strongly disagree (1) to strongly agree (4), and the composite score was computed by taking the mean across all four items (α = 0.85). Seven items assessing perceptions of intergroup interactions in the neighborhood were adapted from the School Climate for Diversity Scale (Byrd, 2017). Two subscales were included: perceptions of quality of intergroup interactions (three items, e.g., “people of different races/ethnicities trust each other”) and perceptions of differential treatment across racial groups, termed intergroup equality (four items, e.g., “people of all races/ethnicities are treated equally in your neighborhood”). All items were measured on a 4‐point Likert scale from strongly disagree (1) to agree (4) and the composite scores were computed by taking the mean across all items (intergroup quality α = 0.71; intergroup equality α = 0.84). Residents' sense of safety was measured by asking whether they typically feel safe in their neighborhood during different times and places (e.g., at night in building hallways), adapted from various sources (e.g., Earls et al., 2007; Rosenbaum et al., 1998). The measure includes four items, asked on a 4‐point Likert scale from strongly disagree (1) to strongly agree (4). A mean composite score was created across all four items (α = 0.80). Finally, the neighborhood problems scale asked residents about perceived problems in their neighborhood and included two subscales: (1) social disorder (eight items) and (2) drugs and violence (seven items) based on Curley (2010) and Earls et al. (2007). Items were scored on a 3‐point scale ranging from not a problem (0) to a big problem (2) and mean composite scores were computed (social disorder, α = 0.76; drugs and violence α = 0.87). The latent profile analysis used standardized versions of all indicators.

#### Individual and contextual factors

Participants reported on ethnicity and race, categorized into the following: Asian, Black, Latino, White, and Other (including Multiracial). Household income was measured using administrative data from the local housing authority, which was then converted into an income‐to‐needs ratio. Subjective social status was measured to capture participant's perceptions of their relative rank within the neighborhood using the MacArthur measure (Adler et al., 2000), a 10‐rung ladder in which the highest rung represents those with the most money, most education, and best jobs, and the bottom rung represents those with the lowest. Gender was self‐reported, with one equal to female, and zero equal to male or nonbinary (one respondent identified as nonbinary; rather than dropping this participant, we include them within the category of “not female”). Age was calculated based on self‐reported birth date. A dichotomous variable measured whether the household included children younger than 18. Participants reported the number of years they have lived in the development (housing tenure). Finally, housing type was a binary variable indicating residence in a townhouse or a multistory apartment building. Townhomes, assigned by lottery with priority going towards larger families, have private entrances, are more spacious, and are organized in small courtyards with lawns in the middle, adjacent to the apartment buildings, which contain far more units, and are organized around larger, often paved courtyards. Unit type may convey a different type of social environment and status (per anecdotes from residents), which could affect social relationships.

#### Mental health outcomes

Participants reported on seven survey items assessing anxiety symptoms in the prior 2 weeks using the General Anxiety Disorder‐7 (GAD‐7) questionnaire (Spitzer et al., 2006). Items were scored on a 4‐point Likert scale from 0 (*not at all*) to 3 (*nearly every day*). A dichotomous variable was created to delineate individuals at or above the cut‐off score of eight, indicating moderate to severe anxiety symptoms (Kroenke et al., 2007). Participants also reported on a seven‐item depression symptom assessment using the same 4‐point Likert scale from 0 (*not at all*) to 3 (*nearly every day*) and 2‐week reference period (α = 0.90) with items drawn from the Patient Health Questionnaire‐8 (PHQ‐8) (Kroenke et al., 2009). One item (“moving or speaking too slowly so that other people could have noticed…”) was removed because it was found to be confusing to participants in pilot tests. After prorating to equate to the original eight‐item scale, we created a dichotomous indicator with a cut‐off score of 10 to identify individuals displaying moderate to severe depressive symptoms (Kroenke et al., 2009). For simplicity, we refer to these measures as respondents showing anxiety and depression, while acknowledging that these are self‐reported symptoms rather than clinical diagnoses.

### Analytic strategy

To examine how public housing residents view their neighborhood social environment, we used Latent Profile Analysis (LPA). LPA is a person‐centered analytic approach used to classify underlying profiles, or sub‐groups within a heterogeneous population. LPA assumes that data are not drawn from a population with a single probability distribution, but rather from a population with a mix of separate distributions, such that an individual's level of the measured variables (i.e., social environment factors) depends on the categorical, latent (unmeasured) grouping variable (Nylund‐Gibson & Choi, 2018; Weller et al., 2020). LPA is ideal for this study because, rather than being interested in the mean level or variance in any single indicator of social environment perceptions, we seek to identify the relationship between multiple theoretically important aspects of the neighborhood social environment. Model building was conducted iteratively, starting with a 1‐class model and adding additional classes one at a time. Consistent with best practice recommendations (Nylund‐Gibson & Choi, 2018; Sinha et al., 2021; Weller et al., 2020), the final model was selected using a multipronged approach, including assessment of parsimony and clustering fit indices, as well as face‐validity of the different solutions (e.g., attention to profile sizes, practical and theoretical relevance, and uniqueness of different profiles). Missing data on neighborhood social environment indicators were addressed using full information maximum likelihood.

To examine potential sources of within‐neighborhood variation in social environment profiles, we first used modal profile assignment to place participants into profiles and conducted bivariate analyses to assess individual and contextual characteristics across profiles. Next, we estimated multinomial regression models predicting profile membership as a function of individual and contextual factors using the Vermunt 3‐step approach to account for uncertainty in profile assignment (Nylund‐Gibson et al., 2014; Vermunt, 2010). The Vermunt 3‐step approach involves (1) estimating the unconditional LPA model, (2) assigning participants to profiles using modal class assignment, and (3) estimating a multinomial model in which model parameters are fixed at values to account for measurement error in profile assignment. Multiple imputation was used to impute missing data on individual and contextual correlates when estimating the multinomial regression models. Finally, the Vermunt 3‐step approach was used to model the relation between profile membership and distal mental health outcomes (anxiety and depression).

## RESULTS

### Descriptive statistics and correlations

Perceptions of the neighborhood social environment were fairly positive, with a mean of around 2.5 to 3 out of 4 (i.e., “somewhat agree”) (Table 1). Residents reported moderate place attachment (*M* = 2.8, SD = 0.9) and perceptions of intergroup interaction quality (*M* = 3.0, SD = 0.7) but were somewhat less likely to agree that all racial‐ethnic groups were treated fairly in the development (intergroup equality, *M* = 2.8, SD = 0.9). Perceptions of neighborhood safety were lower (*M* = 2.6, SD = 0.9), as were perceptions of social cohesion (*M* = 2.5, SD = 0.8) when compared to the other social environment indicators. Residents indicated that social, drug and violence related concerns were “somewhat of a problem,” with drug and violence problems generally rated as more of a problem (*M* = 0.9, SD = 0.6) than social issues (*M* = 0.6, SD = 0.5). Correlations between the social environmental indicators ranged from −0.18 (quality of intergroup interactions and drugs and violence) to 0.69 (neighborhood social problems and drugs and violence, as shown in Table A1).

**Table 1:** Neighborhood social environment indicators (overall and by race).

	Full sample	White	Black	Hispanic or Latino	Asian	Other
Place Attachment	2.85	2.71	2.90	2.83	3.03	2.72
	(0.91)	(0.99)	(0.83)	(1.00)	(0.58)	(0.84)
Social Cohesion	2.54	2.41	2.51	2.53	2.80	2.53
	(0.81)	(0.88)	(0.73)	(0.91)	(0.53)	(0.72)
Neighborhood Intergroup Interactions (Quality)	3.01	2.93	3.05	3.03	2.85	3.13
	(0.70)	(0.71)	(0.68)	(0.79)	(0.55)	(0.69)
Neighborhood Intergroup Interactions (Equal Treatment)	2.80	2.68	2.72	2.82	3.08	2.72
	(0.87)	(0.89)	(0.85)	(0.93)	(0.58)	(0.75)
Neighborhood Safety	2.62	2.50	2.75	2.57	2.75	2.65
	(0.85)	(0.83)	(0.76)	(0.94)	(0.66)	(0.79)
Neighborhood Social Problems	0.58	0.70	0.58	0.60	0.42	0.69
	(0.45)	(0.47)	(0.42)	(0.47)	(0.38)	(0.41)
Neighborhood Problems: Drugs & Violence	0.81	0.99	0.73	0.78	0.52	1.06
	(0.61)	(0.55)	(0.60)	(0.61)	(0.57)	(0.59)

*Note*: Means, with standard deviations in parentheses. Place attachment, social cohesion, neighborhood intergroup interactions, and neighborhood safety measures range from 1 to 4, neighborhood problems measures range from 0 to 2.

### Latent profile analysis of social environmental perceptions

LPA models with one to six profiles were estimated. Considering fit statistics and practical considerations (Nylund‐Gibson & Choi, 2018; Sinha et al., 2021; Weller et al., 2020), a 5‐profile model was the best fit (Table A2). The 5‐profile model had five distinct profiles showing unique mixes of social environment indicators, with each profile representing at least 15% of the sample, and had high entropy (a composite indicator of the degree of separation between classes, wherein values closer to 1 indicate higher separation).

**Figure 1:**
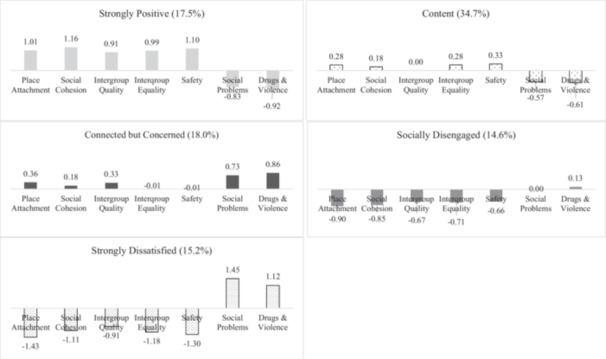
Five latent profiles of social environment perceptions.

Figure 1 shows the mean scores on all indicators for each of the five profiles, using the within‐sample standardized version of all variables such that a score of 0 is equal to the sample average. The largest profile, which we refer to as the ***Content*** profile, represented 35% of the sample and was characterized by just above average scores for social cohesion, place attachment, safety perceptions, and intergroup quality and equality, with below average scores on perceptions of neighborhood social problems and drugs/violence. Another 18% of the sample was ***Strongly Positive***. These residents thought highly of their neighborhood. They were approximately one SD above average on the neighborhood social interaction indicators, including perceptions of safety, and nearly one SD below average on the neighborhood problems scale. The ***Connected but Concerned*** group also comprised 18% of the sample. This profile was above average in terms of place attachment, social cohesion, and intergroup quality, close to the average on safety and intergroup equality, but well above average in terms of perceptions of neighborhood problems. The final two groups each made up 15% of the sample and held more negative perceptions of the neighborhood social environment. The ***Socially Disengaged*** group was 0.66 to 0.90 *SDs* below average on all neighborhood social interaction indicators and perceptions of safety but scored approximately average on the neighborhood problems scales. Lastly, the ***Strongly Dissatisfied*** group scored well below average (0.91 to 1.4 *SDs*) on all neighborhood social interaction and safety indicators and strongly above average on perceptions of neighborhood problems.

### Socio‐demographic factors and latent profile membership

Table 2 shows the demographic characteristics of each profile. Table 3 shows the statistical significance of profile differences based on multinomial models estimated using the Vermunt 3‐step approach, in which the largest profile, ***Content***, was the reference group (notes in Table 3 indicate the significance of other group differences, and the full multinomial model output is shown in Table A3).

**Table 2:** Social demographic characteristics of the 5 latent profiles.

	Sample *M* (SD)	Strongly positive	Content	Connected but concerned	Socially disengaged	Strongly dissatisfied
Asian	15%	11%	29%	11%	4%	5%
Black	19%	15%	24%	21%	21%	11%
Latino	38%	50%	32%	26%	41%	48%
Other	8%	7%	3%	15%	16%	5%
White	19%	17%	13%	27%	17%	30%
Female	66%	71%	57%	63%	74%	76%
Household has children less than 18	36%	32%	31%	40%	39%	46%
Household Income to Needs Ratio	1.28 (1.03)	1.34	1.40	1.23	1.12	1.16
Subjective Social Status: Neighborhood	5.87 (2.28)	6.62	5.78	5.77	5.58	5.66
Age	44.24 (19.36)	49.63	45.82	38.47	41.81	43.26
Tenure	14.44 (10.65)	14.55	15.70	14.36	11.68	14.28
Townhome	23%	29%	23%	17%	23%	19%
*N*	527	92	183	95	77	80

**Table 3:** Relation between individual and contextual characteristics and neighborhood social environment profile membership.

	Strongly positive			Connected but concerned			Socially disengaged			Strongly dissatisfied		
	95% CI			95% CI			95% CI			95% CI		
	Estimate	Lower 2.5%	Upper 2.5%	Estimate	Lower 2.5%	Upper 2.5%	Estimate	Lower 2.5%	Upper 2.5%	Estimate	Lower 2.5%	Upper 2.5%
Asian	**0.177 (0.125)**	**0.045**	**0.703**	**0.130 (0.086)**	**0.036**	**0.475**	0.112 (0.186)	0.004	2.919	**0.048 (0.045)**	**0.008**	**0.306**
Black^a^	0.466 (0.289)	0.138	1.572	**0.252 (0.152)**	**0.077**	**0.824**	0.754 (0.543)	0.184	3.089	**0.125 (0.084)**	**0.034**	**0.463**
Latino^b^	1.114 (0.624)	0.372	3.337	**0.314 (0.172)**	**0.107**	**0.919**	1.26 (0.856)	0.333	4.768	0.587 (0.273)	0.236	1.461
Other^c^	8.449 (18.476)	0.116	614.058	4.719 (9.48)	0.092	241.928	19.598 (41.93)	0.296	1298.335	1.367 (2.769)	0.026	72.496
Female	1.967 (0.851)	0.842	4.592	1.613 (0.683)	0.704	3.699	**3.341 (1.825)**	**1.146**	**9.744**	**2.292 (0.962)**	**1.006**	**5.219**
Household with children	1.029 (0.504)	0.394	2.688	0.941 (0457)	0.364	2.437	0.74 (0.39)	0.263	2.079	1.318 (0.579)	0.558	3.116
Age^d^	1.03 (0.016)	1	1.061	0.977 (0.013)	0.953	1.002	1.007 (0.016)	0.977	1.038	0.991 (0.013)	0.966	1.017
Income to needs ratio	0.957 (0.132)	0.729	1.255	0.861 (0.15)	0.612	1.21	0.802 (0.226)	0.462	1.392	0.766 (0.213)	0.445	1.32
Subjective Social Status^e^	1.15 (0.095)	0.978	1.353	1.027 (0.083)	0.877	1.203	0.943 (0.09)	0.781	1.138	0.976 (0.09)	0.814	1.17
Tenure^f^	0.967 (0.018)	0.931	1.004	1.004 (0.02)	0.965	1.045	**0.949 (0.024)**	**0.904**	**0.996**	0.998 (0.02	0.96	1.038
Townhome^g^	2.045 (0.98)	0.799	5.233	0.495 (0.28)	0.164	1.501	1.85 (0.952)	0.675	5.071	0.627 (0.302)	0.244	1.611

*Note*: Multinomial models estimated using the Vermunt 3‐step Approach to adjust for uncertainty in profile membership. Bold text indicates a statistically significant association. Reference group is the Content profile. Findings when using other profiles as the reference group are indicated via subscripts.

^a^ Black residents significantly less likely than White residents to be Dissatisfied versus Socially Disengaged.

^b^ Latino residents significantly less likely than White residents to be Connected but Concerned versus Strongly Positive or Socially Disengaged.

^c^ Residents from other racial groups significantly less likely than White residents to be Strongly Dissatisfied versus Socially Disengaged.

^d^ Age positively associated with membership in Strongly Positive versus Connected but Concerned or Strongly Dissatisfied.

^e^ Neighborhood SSS positively associated with membership in Strongly Positive versus Socially Disengaged.

^f^ Tenure in the development positively associated with membership in Connected but Concerned versus Socially Disengaged.

^g^ Townhome residents more likely than apartment building residents to be Strongly Positive versus Strongly Dissatisfied or Connected but Concerned.

In terms of race, Asian residents were concentrated in the ***Content*** profile. Black residents were roughly equally spread between all profiles except for a lower prevalence in the ***Strongly Dissatisfied*** profile. Latino residents were concentrated in the two extremes—***Strongly Positive***, or ***Strongly Dissatisfied***, with a lower prevalence in the ***Connected but Concerned*** profile. White residents were predominantly in the ***Connected but Concerned*** and ***Strongly Dissatisfied*** groups. Multinomial models indicate that these general patterns of racial differences in profile membership were statistically significant after accounting for all other predictors and for uncertainty in profile membership.

In terms of gender, female residents were significantly more likely to be either ***Socially Disengaged*** or ***Strongly Dissatisfied*** versus ***Content***. Households with children were disproportionately in the ***Strongly Dissatisfied*** group (vs. ***Content***), but this difference was not statistically significant in multinomial models. Regarding socioeconomic factors, household income was highest in the ***Content*** profile, while subjective social status was highest in the ***Strongly Positive*** profile, and both were lowest in the ***Socially Disengaged*** profile, a significant difference for subjective social status only. Age was highest in the ***Strongly Positive*** and lowest in the ***Connected but Concerned*** groups, statistically significant differences.

Finally, with regard to housing type, residents living in townhomes (as compared to multi‐story apartment buildings) were most likely to be in the ***Strongly Positive*** group and least likely to be in the ***Connected but Concerned*** and ***Strongly Dissatisfied*** groups, and these differences were statistically significant. Housing tenure (time living in the development) was lowest in ***Socially Disengaged*** and highest in the ***Content*** group, also a statistically significant difference (Table 3).

### Relationships between social environment perceptions and health

**Figure 2:**
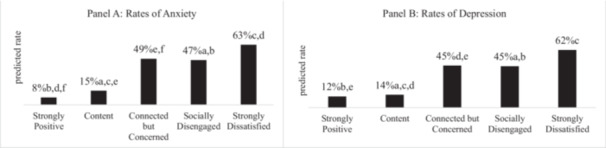
Rates of anxiety and depression, by social environment profile. *Note*: Predicted rates of anxiety and depressive based on profile membership, estimated using the Vermunt 3‐step approach to adjust for uncertainty in profile membership. Within each panel, predicted rates with the same subscript are statistically different at *p *< 0.05 (see chi‐square test statistics in Table A4).

Our final analytic step assessed rates of anxiety and depression across the five social environment profiles, estimated using the Vermunt 3‐step approach. As shown in Figure 2, rates of anxiety and depression were lowest in the ***Strongly Positive*** (8% and 12%, respectively) and ***Content*** profiles (15% and 14%), with both significantly lower than the other three profiles (chi‐square test statistics and *p*‐values are shown in Table A4). The highest rates of anxiety and depression were seen in the ***Strongly Dissatisfied*** profile (63% and 62%), with rates 4 to 5 times higher than the former two profiles. These were followed by the ***Socially Disengaged*** and ***Connected but Concerned*** profiles, with rates of anxiety and depression between 45% and 49%. It is notable that these two profiles had similar rates of anxiety and depression, because to some extent they reflect opposite perceptions of the social environment—either low levels of social engagement and low perceived problems (***Socially Disengaged***), or strong levels of social engagement but high perceived problems (***Connected but Concerned***).

## DISCUSSION

Neighborhoods are a critical context shaping youth and adult mental health. Despite growing attention to the role of social processes in driving the link between neighborhoods and mental health, few studies have examined the clustering of multiple, co‐occurring neighborhood strengths and risk factors. Further, little prior attention has been paid to how individuals' perceptions of neighborhood social environments differ *within* (rather than between) neighborhoods to help explain individual differences in health and to highlight not only challenges but also strengths of underserved communities. This study addressed these gaps by examining patterns of intergroup interactions, social cohesion, place attachment, sense of safety, and neighborhood problems within a single large and racially diverse public housing development. Findings show substantial intra‐neighborhood variability in social environment perceptions. This variability was systematically related to individual and contextual factors and associated with large differences in rates of anxiety and depression.

We found five sub‐groups, or profiles of residents with unique perceptions of the neighborhood social environment. Two groups had positive perceptions; one extremely so (***Strongly Positive***, 18% of the sample), and one that agreed at least somewhat that social interactions were strong, and drugs/violence and other social issues were not at all or only somewhat of a problem (***Content***, 35% of the sample). A third group reported strong social ties to the neighborhood but was concerned about social problems and drugs and violence (***Connected but Concerned***, 18%). Representing a contrasting viewpoint, the ***Socially Disengaged*** group had weak neighborhood social ties, yet was not concerned about social problems or drugs and violence (***Socially Disengaged***, 15%). The fifth group was not well integrated and highly concerned about neighborhood problems (***Strongly Dissatisfied***, 15%).

The majority of residents reported positive perceptions of the quality of cross‐race interactions—including trust between members of different racial groups. This finding runs counter to social disorganization theory (Sampson & Groves, 1989), which posits that structurally disadvantaged and racially diverse neighborhoods will spur competition for scarce resources and mistrust between neighbors. The unique context of this study may provide real‐world support for the four components of intergroup contact theory (Allport, 1954; Pettigrew, 1998). Racial groups in the development have relatively equal economic status, and in some sense, they can be described as working towards the common goal of making their community livable despite the deteriorating physical conditions of the development, and despite the violence and crime that does occur. The environment is also noncompetitive, in that all residents have gained a spot in public housing. However, the fourth condition of Allport's model—support for intergroup contact by authorities—may not apply in this context. Study participants agreed only somewhat that neighborhood authorities (police, the housing authority) treated residents of different racial groups equally (though equal treatment is not the same thing as support for positive intergroup contact, which was not directly measured in this study).

Moreover, perceptions of the neighborhood social environment varied across racial groups. Latino residents—the largest racial group in the development, representing 38% of the study sample—were concentrated in two very different profiles: ***Strongly Dissatisfied*** and ***Strongly Positive***. This shows the heterogeneity of neighborhood experiences within the same neighborhood and the *same racial group*. Somewhat surprisingly considering the history of anti‐Black racism in the neighborhood (Dominguez, 2012), Black residents were significantly less likely to be in the ***Strongly Dissatisfied*** profile compared to White residents. Asian residents were disproportionately in the ***Content*** group, which is also somewhat unexpected considering Asian residents are the most recent arrivals to the neighborhood and English proficiency is lowest among Asians compared to other racial groups in the study neighborhood. Patterns of racial differences may be explained in part by different historical experiences in the community, diverse cultural and language norms, and immigration experiences, all of which are important issues to explore in future research.

Neighborhood perceptions also varied across gender, age, and building type. Females were significantly more likely to be ***Socially Disengaged*** or ***Strongly Dissatisfied***, potentially related to safety concerns that disproportionately affect women and girls. Older residents were significantly more likely than younger residents to be ***Strongly Positive***, whereas younger residents were more likely to be ***Connected but Concerned*** or ***Strongly Dissatisfied***. Older residents may have stronger social ties to the extent that they have lived longer in the neighborhood, while youth may be well integrated socially (especially if they attend the same schools). However, youth also likely have greater exposure to drugs and violence in the neighborhood, issues which tend to involve youth more than older adults (Goldstick et al., 2018). Residents in town homes were also more likely to be in the ***Strongly Positive*** group (vs. ***Strongly Dissatisfied***) compared to those in multistory apartment buildings. Future work should unpack what it is about the townhomes that facilitates positive social ties and decreased safety concerns. The larger size and private entrance of the townhomes could provide a positive lens through which residents view their neighborhood. In addition, the townhome's grassy central courtyards might provide a more comfortable space for interacting with neighbors than the concrete courtyards and stairways of the multistory apartment buildings, in which drug use and squatting by non‐residents often occurred. As the development prepares for redevelopment into a mixed‐income community, these findings show the need to tailor neighborhood social programming to meet the unique needs and strengths of different sub‐populations, and to consider how different design choices—such as townhomes versus apartment buildings—affect residents' social interactions and sense of safety.

Consistent with prior evidence focused on specific social processes in isolation (Browning et al., 2008; Hong et al., 2014; Kim, 2010; Rios et al., 2012; Scannell & Gifford, 2017), rates of anxiety and depression were lowest in the ***Strongly Positive*** profile, followed closely by the ***Content*** profile, and highest‐ approximately four to five times higher‐ in the ***Strongly Dissatisfied*** profile. However, rates of anxiety and depression were also substantially elevated in the two profiles that contained a mix of both positive and negative perceptions (***Connected but Concerned*** and ***Socially Disengaged***). This finding shows how multiple, co‐occurring neighborhood stressors and strengths together may influence mental health, a unique result which would not have been possible through a variable‐centered approach where stressors and strengths are examined individually. The observed association between profile membership and mental health also strengthens understanding of social determinants of health by delineating how differences *within* (not just between) neighborhoods relate to health outcomes. However, it is important to acknowledge that our results are correlational and cross‐sectional and do not imply causality. It is possible that perceptions of the neighborhood social environment affect mental health, or vice versa. For example, anxiety or depression may cause people to withdraw from their neighborhood, leading to less positive perceptions of the social environment.

### Policy implications

One of the main findings from research on prior public housing redevelopments is that public housing residents who remained post‐redevelopment reported a decreased sense of belonging as the neighborhood transitioned from being 100% public housing residents to mixed‐income (e.g., Chaskin et al., 2012, 2013; McCormick et al., 2012). The findings presented herein demonstrate the importance of maintaining and strengthening existing social networks among public housing residents and of connecting residents across race, gender, and social class. Maintaining social networks will be key for those in profiles with positive connections, and strengthening social networks could contribute to improved outcomes for those who are disconnected, dissatisfied, or mixed.

### Limitations and areas for future research

This study has several limitations. Data were drawn from a racially diverse sample of public housing residents—a group which is underexamined in neighborhood research despite its theoretical and policy import—and from a broad range of ages. However, as noted, the cross‐sectional and correlational nature of the data precludes us from determining directionality and causality in the relationship between social environment perceptions and mental health and the profiles identified are specific to the current sample. We also note that what we observed are within‐neighborhood differences in neighborhood *perceptions*. Future work should examine how self‐reported perceptions of the neighborhood social environment map on to other objectively measured features of within‐neighborhood variation (e.g., racial segregation between buildings, proximity to resources and green space, and exposure to crime or disorder). In addition, the measures of intergroup interactions used in this study were limited in that they focused on general perceptions of race relations, whereas measures that ask about how one's own racial group is treated within the neighborhood may better capture experiences of discrimination and prejudice. It is also important to note that we left the definition of neighborhood up to respondents, who may have responded in reference to the broader neighborhood, the housing development, their specific building or street within the development, or some combination thereof. This choice is consistent with evidence showing that people use different geographic boundaries to define their neighborhoods and these definitions rarely align with researcher‐defined measures like census tracts (Burdick‐Will, 2018; Pratt et al., 2020). Finally, future work should also examine differences in social environment perceptions related to intersections of gender, race, age, and other status‐based identities (e.g., disability or immigration).

## CONCLUSION

This study examined the relationship between multiple, co‐occurring neighborhood‐level social processes and youth and adult mental health. We found rich variability in patterns of cross‐race interactions, social cohesion, place attachment, and neighborhood problems within a single large and racially diverse public housing development. This variability was systematically related to individual and contextual factors and strongly associated with mental health outcomes, with implications for the tailoring of neighborhood social programs to meet the unique needs and strengths of public housing residents.

## AUTHOR CONTRIBUTIONS

**Jane Leer**: Conceptualization, data curation, formal analysis, methodology, project administration, writing—original draft, and writing—review and editing. **Lindsay Lanteri**: Data curation, formal analysis, writing—original draft, and writing—review and editing. **Samantha Teixeira**: Supervision, writing—review and editing, funding acquisition. **Rebekah Levine Coley**: Supervision, methodology, writing—review and editing, funding acquisition.

## CONFLICT OF INTEREST STATEMENT

The authors declare no conflicts of interest.

## ETHICS STATEMENT

The manuscript reports on data that were collected in compliance with APA ethical standards, under the approval of the Boston College Institutional Review Board. Parent/guardian consent was obtained for all minors.

## Supporting information

Supporting information.

## Data Availability

The data that support the findings of this study are not publicly available.
