# Supplementary material for: Neighborhood social environments and mental health among youth and adults in public housing
Source: Am J Community Psychol. 2025 Nov 28;77(3-4):413–26. doi: 10.1002/ajcp.70036 (PMC13289514; doi:10.1002/ajcp.70036)
Supplement: Supplementary file 1 — Supporting information. [file AJCP-77-413-s001.docx]

**Appendix A**

**Additional Tables**

**Table A1. Panel A Correlations between social environment indicators and individual characteristics**

|  |  | (1) | (2) | (3) | (4) | (5) | (6) | (7) |
| --- | --- | --- | --- | --- | --- | --- | --- | --- |
| (1) | Place Attachment | 1.00 |  |  |  |  |  |  |
| (2) | Social Cohesion | 0.62 | 1.00 |  |  |  |  |  |
| (3) | Intergroup Quality | 0.50 | 0.56 | 1.00 |  |  |  |  |
| (4) | Intergroup Equality | 0.54 | 0.61 | 0.57 | 1.00 |  |  |  |
| (5) | Neighborhood Safety | 0.66 | 0.55 | 0.48 | 0.51 | 1.00 |  |  |
| (6) | Neighborhood Social Problems | -0.44 | -0.44 | -0.29 | -0.52 | -0.50 | 1.00 |  |
| (7) | Neighborhood Problems: Drugs and Violence | -0.43 | -0.37 | -0.18 | -0.40 | -0.52 | 0.69 | 1.00 |
| (8) | Asian | 0.10 | 0.15 | -0.09 | 0.15 | 0.07 | -0.19 | -0.20 |
| (9) | Black | 0.03 | -0.03 | 0.04 | -0.05 | 0.08 | -0.03 | -0.07 |
| (10) | Latino | -0.02 | -0.01 | 0.03 | 0.02 | -0.05 | 0.02 | -0.03 |
| (11) | Other | -0.04 | -0.01 | 0.08 | -0.04 | 0.01 | 0.05 | 0.15 |
| (12) | White | -0.07 | -0.09 | -0.05 | -0.08 | -0.08 | 0.12 | 0.17 |
| (13) | Female | -0.05 | 0.03 | -0.02 | -0.10 | -0.13 | 0.05 | 0.04 |
| (14) | Household has children less than 18 | -0.11 | -0.05 | 0.01 | -0.04 | -0.11 | 0.07 | 0.11 |
| (15) | Household income to needs ratio | 0.09 | 0.03 | -0.02 | 0.02 | 0.05 | -0.04 | -0.08 |
| (16) | Subjective social status: neighborhood | 0.09 | 0.13 | 0.13 | 0.13 | 0.15 | -0.11 | -0.10 |
| (17) | Age | 0.12 | 0.10 | -0.03 | 0.07 | 0.11 | -0.11 | -0.20 |
| (18) | Years lived in development | 0.11 | 0.06 | 0.01 | 0.01 | 0.06 | 0.03 | -0.04 |
| (19) | Lives in a row home | 0.07 | 0.00 | -0.05 | 0.03 | -0.02 | -0.09 | -0.11 |

**Table A1. Panel B: Correlations between individual characteristics**

|  |  | | (8) | (9) | (10) | (11) | (12) | (13) | (14) | (15) | (16) | (17) | (18) | (19) |
| --- | --- | --- | --- | --- | --- | --- | --- | --- | --- | --- | --- | --- | --- | --- |
| (8) | | Asian | 1.00 |  |  |  |  |  |  |  |  |  |  |  |
| (9) | | Black | -1.00 | 1.00 |  |  |  |  |  |  |  |  |  |  |
| (10) | | Latino | -1.00 | -1.00 | 1.00 |  |  |  |  |  |  |  |  |  |
| (11) | | Other | -1.00 | -1.00 | -1.00 | 1.00 |  |  |  |  |  |  |  |  |
| (12) | | White | -1.00 | -1.00 | -1.00 | -1.00 | 1.00 |  |  |  |  |  |  |  |
| (13) | | Female | -0.17 | -0.04 | 0.19 | -0.19 | 0.03 | 1.00 |  |  |  |  |  |  |
| (14) | | Household has children less than 18 | -0.23 | 0.21 | 0.09 | 0.15 | -0.23 | 0.38 | 1.00 |  |  |  |  |  |
| (15) | | Household income to needs ratio | 0.03 | 0.03 | -0.04 | -0.05 | 0.02 | -0.02 | -0.06 | 1.00 |  |  |  |  |
| (16) | | Subjective social status: neighborhood | -0.10 | 0.02 | 0.19 | -0.03 | -0.14 | 0.01 | 0.04 | 0.07 | 1.00 |  |  |  |
| (17) | | Age | 0.15 | -0.15 | -0.01 | -0.17 | 0.15 | 0.06 | -0.53 | 0.00 | 0.02 | 1.00 |  |  |
| (18) | | Years lived in development | 0.16 | -0.21 | -0.07 | -0.08 | 0.21 | 0.02 | 0.02 | 0.15 | -0.05 | 0.37 | 1.00 |  |
| (19) | | Lives in a row home | 0.15 | -0.23 | 0.15 | -0.52 | 0.05 | 0.09 | -0.03 | 0.12 | -0.02 | -0.14 | 0.22 | 1.00 |

**Table A2. Fit Statistics for Profile Models**

|  | |  | |  | |  | | | Parsimony Criteria | | | | Clustering Criteria | | | | Both |
| --- | --- | --- | --- | --- | --- | --- | --- | --- | --- | --- | --- | --- | --- | --- | --- | --- | --- |
| Classes | N | | parms | | LL | | Entropy | AIC | | BIC | CAIC | ssBIC | | CLC | NEC | E | ICL-BIC |
| 1 | 527 | | 14 | | -4705.2 | | 0 | 9438.4 | | 9498.14 | 9512.14 | 9453.7 | | 9410.4 | 1 | NA | 9498.14 |
| 2 | 527 | | 22 | | -4180.65 | | 52.9668 | 8405.29 | | 8499.17 | 8521.17 | 8429.34 | | 8467.23 | 0.10098 | 0.855 | 8605.1 |
| 3 | 527 | | 30 | | -4055.54 | | 125.057 | 8171.08 | | 8299.1 | 8329.1 | 8203.87 | | 8361.2 | 0.1925 | 0.784 | 8549.21 |
| 4 | 527 | | 38 | | -3948.63 | | 176.069 | 7973.26 | | 8135.42 | 8173.42 | 8014.79 | | 8249.4 | 0.23272 | 0.759 | 8487.55 |
| 5 | 527 | | 46 | | -3948.63 | | 170.483 | 7989.26 | | 8185.55 | 8231.55 | 8039.54 | | 8238.23 | 0.775 | 0.799 | 8526.52 |
| 6 | 527 | | 54 | | -3904.08 | | 184.13 | 7916.15 | | 8146.58 | 8200.58 | 7975.17 | | 8176.41 | 0.66577 | 0.805 | 8514.84 |

**Table A3. Relation between individual and contextual characteristics and social environment profile membership**

| **Panel A. Reference Group: Strongly Positive** | | | | |  |  |  |  |  |  |  |  |  |  |  |  |
| --- | --- | --- | --- | --- | --- | --- | --- | --- | --- | --- | --- | --- | --- | --- | --- | --- |
|  | **Content** | | | | **Connected but Concerned** | | | | **Socially Disengaged** | | | | **Dissatisfied** | | | |
|  |  |  | 95% CI | |  |  | 95% CI | |  |  | 95% CI | |  |  | 95% CI | |
|  | Estimate | S.E. | Lower 2.5% | Upper 2.5% | Estimate | S.E. | Lower 2.5% | Upper 2.5% | Estimate | S.E. | Lower 2.5% | Upper 2.5% | Estimate | S.E. | Lower 2.5% | Upper 2.5% |
| Asian | **5.636** | **3.96** | **1.422** | **22.335** | 0.732 | 0.607 | 0.144 | 3.717 | 0.631 | 1.024 | 0.026 | 15.192 | 0.271 | 0.298 | 0.031 | 2.335 |
| Black | 2.146 | 1.331 | 0.636 | 7.239 | 0.54 | 0.342 | 0.156 | 1.865 | 1.619 | 1.153 | 0.401 | 6.542 | 0.268 | 0.195 | 0.065 | 1.113 |
| Latino | 0.897 | 0.502 | 0.3 | 2.688 | **0.282** | **0.149** | **0.1** | **0.792** | 1.131 | 0.728 | 0.32 | 3.992 | 0.527 | 0.251 | 0.208 | 1.338 |
| Other | 0.118 | 0.259 | 0.002 | 8.601 | 0.559 | 0.436 | 0.121 | 2.575 | 2.32 | 1.967 | 0.44 | 12.228 | 0.162 | 0.156 | 0.025 | 1.067 |
| Female | 0.508 | 0.22 | 0.218 | 1.187 | 0.82 | 0.366 | 0.342 | 1.967 | 1.699 | 0.896 | 0.604 | 4.779 | 1.165 | 0.537 | 0.472 | 2.878 |
| Household with children | 0.972 | 0.476 | 0.372 | 2.539 | 0.915 | 0.455 | 0.345 | 2.425 | 0.719 | 0.353 | 0.274 | 1.885 | 1.281 | 0.609 | 0.504 | 3.255 |
| Age | 0.971 | 0.015 | 0.942 | 1 | **0.949** | **0.014** | **0.922** | **0.977** | 0.978 | 0.015 | 0.948 | 1.008 | **0.962** | **0.015** | **0.934** | **0.992** |
| Income to needs ratio | 1.045 | 0.145 | 0.797 | 1.371 | 0.9 | 0.166 | 0.627 | 1.292 | 0.838 | 0.23 | 0.489 | 1.436 | 0.801 | 0.235 | 0.45 | 1.425 |
| Subjective social status | 0.869 | 0.072 | 0.739 | 1.023 | 0.893 | 0.075 | 0.758 | 1.052 | **0.82** | **0.075** | **0.684** | **0.982** | 0.848 | 0.08 | 0.705 | 1.021 |
| Tenure | 1.034 | 0.02 | 0.996 | 1.074 | 1.039 | 0.02 | 1 | 1.079 | 0.982 | 0.024 | 0.936 | 1.029 | 1.033 | 0.022 | 0.991 | 1.076 |
| Townhome | 0.489 | 0.234 | 0.191 | 1.251 | **0.242** | **0.129** | **0.086** | **0.685** | 0.905 | 0.434 | 0.353 | 2.316 | **0.307** | **0.153** | **0.116** | **0.813** |
|  |  |  |  |  |  |  |  |  |  |  |  |  |  |  |  |  |
| **Panel B. Reference Group: Connected but concerned** | | | | | |  |  |  |  |  |  |  |  |  |  |  |
|  | **Strongly Positive** | | | | **Content** | | | | **Dissatisfied** | | | | **Socially Disengaged** | | | |
|  |  |  | 95% CI | |  |  | 95% CI | |  |  | 95% CI | |  |  | 95% CI | |
|  | Estimate | S.E. | Lower 2.5% | Upper 2.5% | Estimate | S.E. | Lower 2.5% | Upper 2.5% | Estimate | S.E. | Lower 2.5% | Upper 2.5% | Estimate | S.E. | Lower 2.5% | Upper 2.5% |
|  |  |  |  |  |  |  |  |  |  |  |  |  |  |  |  |  |
| Asian | 1.366 | 1.133 | 0.269 | 6.939 | **7.701** | **5.092** | **2.107** | **28.146** | 0.37 | 0.359 | 0.055 | 2.474 | 0.862 | 1.568 | 0.024 | 30.448 |
| Black | 1.851 | 1.17 | 0.536 | 6.392 | **3.973** | **2.404** | **1.213** | **13.007** | 0.496 | 0.34 | 0.13 | 1.897 | 2.997 | 2.324 | 0.656 | 13.698 |
| Latino | **3.55** | **1.872** | **1.262** | **9.982** | **3.186** | **1.745** | **1.089** | **9.321** | 1.871 | 0.901 | 0.728 | 4.811 | **4.015** | **2.806** | **1.021** | **15.794** |
| Other | 1.79 | 1.396 | 0.388 | 8.255 | 0.212 | 0.426 | 0.004 | 10.862 | 0.29 | 0.247 | 0.054 | 1.539 | 4.153 | 3.649 | 0.742 | 23.246 |
| Female | 1.219 | 0.544 | 0.508 | 2.923 | 0.62 | 0.262 | 0.27 | 1.421 | 1.42 | 0.627 | 0.598 | 3.372 | 2.071 | 1.212 | 0.658 | 6.52 |
| Household with children | 1.093 | 0.544 | 0.412 | 2.897 | 1.062 | 0.515 | 0.41 | 2.75 | 1.4 | 0.672 | 0.547 | 3.588 | 0.786 | 0.453 | 0.254 | 2.434 |
| Age | **1.054** | **0.016** | **1.023** | **1.085** | 1.023 | 0.013 | 0.998 | 1.049 | 1.014 | 0.014 | 0.987 | 1.041 | 1.03 | 0.017 | 0.998 | 1.064 |
| Income to needs ratio | 1.111 | 0.205 | 0.774 | 1.596 | 1.162 | 0.202 | 0.826 | 1.633 | 0.89 | 0.253 | 0.51 | 1.553 | 0.931 | 0.293 | 0.502 | 1.727 |
| Subjective social status | 1.12 | 0.094 | 0.95 | 1.319 | 0.973 | 0.078 | 0.831 | 1.14 | 0.95 | 0.092 | 0.785 | 1.148 | 0.918 | 0.092 | 0.754 | 1.116 |
| Tenure | 0.963 | 0.019 | 0.927 | 1 | 0.996 | 0.02 | 0.957 | 1.036 | 0.994 | 0.021 | 0.954 | 1.036 | **0.945** | **0.026** | **0.896** | **0.997** |
| Townhome | **4.129** | **2.19** | **1.459** | **11.68** | 2.018 | 1.141 | 0.666 | 6.115 | 1.266 | 0.694 | 0.433 | 3.708 | **3.735** | **2.219** | **1.165** | **11.97** |
|  |  |  |  |  |  |  |  |  |  |  |  |  |  |  |  |  |
|  |  |  |  |  |  |  |  |  |  |  |  |  |  |  |  |  |
| **Panel C. Reference Group: Socially Disengaged** | | | | |  |  |  |  |  |  |  |  |  |  |  |  |
|  | **Strongly Positive** | | | | **Content** | | | | **Connected but Concerned** | | | | **Dissatisfied** | | | |
|  |  |  | 95% CI | |  |  | 95% CI | |  |  | 95% CI | |  |  | 95% CI | |
|  | Estimate | S.E. | Lower 2.5% | Upper 2.5% | Estimate | S.E. | Lower 2.5% | Upper 2.5% | Estimate | S.E. | Lower 2.5% | Upper 2.5% | Estimate | S.E. | Lower 2.5% | Upper 2.5% |
|  |  |  |  |  |  |  |  |  |  |  |  |  |  |  |  |  |
| Asian | 1.584 | 2.571 | 0.066 | 38.131 | 8.929 | 14.854 | 0.343 | 232.71 | 1.159 | 2.108 | 0.033 | 40.934 | 0.429 | 0.91 | 0.007 | 27.375 |
| Black | 0.618 | 0.44 | 0.153 | 2.497 | 1.326 | 0.954 | 0.324 | 5.429 | 0.334 | 0.259 | 0.073 | 1.525 | **0.166** | **0.146** | **0.029** | **0.931** |
| Latino | 0.884 | 0.569 | 0.25 | 3.12 | 0.793 | 0.539 | 0.21 | 3.001 | **0.249** | **0.174** | **0.063** | **0.98** | 0.466 | 0.312 | 0.126 | 1.728 |
| Other | 0.431 | 0.366 | 0.082 | 2.273 | 0.051 | 0.109 | 0.001 | 3.38 | 0.241 | 0.212 | 0.043 | 1.348 | **0.07** | **0.075** | **0.009** | **0.568** |
| Female | 0.589 | 0.311 | 0.209 | 1.656 | 0.299 | **0.163** | **0.103** | **0.873** | 0.483 | 0.283 | 0.153 | 1.521 | 0.686 | 0.435 | 0.198 | 2.379 |
| Household with children | 1.391 | 0.683 | 0.531 | 3.644 | 1.352 | 0.712 | 0.481 | 3.797 | 1.272 | 0.734 | 0.411 | 3.939 | 1.782 | 1.021 | 0.58 | 5.477 |
| Age | 1.023 | 0.016 | 0.992 | 1.055 | 0.993 | 0.015 | 0.963 | 1.024 | 0.971 | 0.016 | 0.94 | 1.002 | 0.984 | 0.018 | 0.95 | 1.019 |
| Income to needs ratio | 1.193 | 0.328 | 0.696 | 2.046 | 1.247 | 0.351 | 0.718 | 2.166 | 1.074 | 0.338 | 0.579 | 1.991 | 0.956 | 0.43 | 0.396 | 2.31 |
| Subjective social status | **1.22** | **0.112** | **1.019** | **1.461** | 1.061 | 0.102 | 0.879 | 1.28 | 1.09 | 0.109 | 0.896 | 1.326 | 1.035 | 0.125 | 0.816 | 1.312 |
| Tenure | 1.019 | 0.025 | 0.972 | 1.068 | **1.054** | **0.026** | **1.004** | **1.106** | 1.058 | **0.029** | **1.003** | **1.117** | 1.052 | 0.031 | 0.992 | 1.115 |
| Townhome | 1.105 | 0.53 | 0.432 | 2.83 | 0.54 | 0.278 | 0.197 | 1.481 | **0.268** | **0.159** | **0.084** | **0.858** | 0.339 | 0.201 | 0.106 | 1.085 |
|  |  |  |  |  |  |  |  |  |  |  |  |  |  |  |  |  |
|  |  |  |  |  |  |  |  |  |  |  |  |  |  |  |  |  |
| **Panel D. Reference Group: Strongly Dissatisfied** | | | | |  |  |  |  |  |  |  |  |  |  |  |  |
|  | **Strongly Positive** | | | | **Content** | | | | **Connected but Concerned** | | | | **Socially Disengaged** | | | |
|  |  |  | 95% CI | |  |  | 95% CI | |  |  | 95% CI | |  |  | 95% CI | |
|  | Estimate | S.E. | Lower 2.5% | Upper 2.5% | Estimate | S.E. | Lower 2.5% | Upper 2.5% | Estimate | S.E. | Lower 2.5% | Upper 2.5% | Estimate | S.E. | Lower 2.5% | Upper 2.5% |
|  |  |  |  |  |  |  |  |  |  |  |  |  |  |  |  |  |
| Asian | 3.691 | 4.057 | 0.428 | 31.82 | **20.806** | **19.646** | **3.269** | **132.43** | 2.702 | 2.618 | 0.404 | 18.055 | 2.33 | 4.94 | 0.037 | 148.62 |
| Black | 3.729 | 2.708 | 0.899 | 15.476 | **8.002** | **5.352** | **2.158** | **29.681** | 2.014 | 1.378 | 0.527 | 7.698 | **6.036** | **5.316** | **1.074** | **33.92** |
| Latino | 1.897 | 0.902 | 0.747 | 4.817 | 1.703 | 0.792 | 0.684 | 4.236 | 0.534 | 0.258 | 0.208 | 1.374 | 2.146 | 1.435 | 0.579 | 7.956 |
| Other | 6.183 | 5.951 | 0.937 | 40.783 | 0.732 | 1.483 | 0.014 | 38.817 | 3.453 | 2.943 | 0.65 | 18.35 | **14.341** | **15.344** | **1.761** | **116.78** |
| Female | 0.858 | 0.396 | 0.348 | 2.12 | **0.436** | **0.183** | **0.192** | **0.994** | 0.704 | 0.311 | 0.297 | 1.672 | 1.458 | 0.925 | 0.42 | 5.056 |
| Household with children | 0.78 | 0.371 | 0.307 | 1.982 | 0.759 | 0.333 | 0.321 | 1.793 | 0.714 | 0.343 | 0.279 | 1.83 | 0.561 | 0.322 | 0.183 | 1.725 |
| Age | **1.039** | **0.016** | **1.009** | **1.071** | 1.009 | 0.013 | 0.983 | 1.035 | 0.986 | 0.014 | 0.96 | 1.013 | 1.016 | 0.018 | 0.981 | 1.053 |
| Income to needs ratio | 1.249 | 0.367 | 0.702 | 2.221 | 1.305 | 0.362 | 0.757 | 2.249 | 1.124 | 0.319 | 0.644 | 1.961 | 1.046 | 0.471 | 0.433 | 2.528 |
| Subjective social status | 1.179 | 0.111 | 0.979 | 1.419 | 1.025 | 0.095 | 0.855 | 1.229 | 1.053 | 0.102 | 0.871 | 1.273 | 0.966 | 0.117 | 0.762 | 1.225 |
| Tenure | 0.968 | 0.02 | 0.93 | 1.009 | 1.002 | 0.02 | 0.963 | 1.041 | 1.006 | 0.021 | 0.965 | 1.048 | 0.951 | 0.028 | 0.896 | 1.008 |
| Townhome | **3.26** | **1.622** | **1.23** | **8.642** | 1.594 | 0.767 | 0.621 | 4.092 | 0.79 | 0.433 | 0.27 | 2.312 | 2.949 | 1.749 | 0.922 | 9.432 |

*Note*: Multinomial models estimated using the Vermunt 3-step Approach to adjust for uncertainty in profile membership.

**Table A4. Chi-square test statistics, difference in anxiety and depression across social environment profiles**

|  | Anxiety | | Depression | | |
| --- | --- | --- | --- | --- | --- |
|  | Chi-2 | P-value | | Chi-2 | P-value |
| Socially Disengaged vs. Strongly Dissatisfied | 1.256 | 0.262 | | 1.603 | 0.206 |
| Socially Disengaged vs. Content | 9.335 | 0.002 | | 9.243 | 0.002 |
| Socially Disengaged vs. Strongly Positive | 15.229 | 0.000 | | 10.616 | 0.001 |
| Socially Disengaged vs. Connected but Concerned | 0.008 | 0.930 | | 0.004 | 0.950 |
| Strongly Dissatisfied vs. Content | 33.913 | 0.000 | | 35.362 | 0.000 |
| Strongly Dissatisfied vs. Strongly Positive | 49.553 | 0.000 | | 0.17 | 0.680 |
| Strongly Dissatisfied vs. Connected but Concerned | 1.994 | 0.158 | | 2.735 | 0.098 |
| Content vs. Strongly Positive | 2.897 | 0.089 | | 0.17 | 0.680 |
| Content vs. Connected but Concerned | 11.272 | 0.001 | | 12.231 | 0.000 |
| Strongly Positive vs. Connected but Concerned | 21.047 | 0.000 | | 15.105 | 0.000 |
